# Exploring the Molecular Functions and Immune Relevance of Macrophage-Associated Genes in Atherosclerosis

**DOI:** 10.1155/humu/9034896

**Published:** 2025-08-20

**Authors:** Chenchen Yu, Haoran Wang, Huiting Xu, Peipei Kang, Jingjing Shao, Hui Zhang

**Affiliations:** ^1^Department of Clinical Laboratory Medicine, Affiliated Tumor Hospital of Nantong University, Nantong, Jiangsu, China; ^2^Department of Immunology, School of Basic Medical Sciences, Nantong University, Nantong, Jiangsu, China; ^3^Department of Anesthesiology, Tumor Hospital Affiliated to Nantong University, Nantong, Jiangsu, China; ^4^Department of Clinical Laboratory Medicine, Tumor Hospital Affiliated to Nantong University, Nantong, Jiangsu, China; ^5^Department of Anesthesiology, First Affiliated Hospital of Soochow University, Suzhou, Jiangsu, China

**Keywords:** atherosclerosis, biomarkers, gene function, immune infiltration, macrophage

## Abstract

Atherosclerosis is a common and significant cardiovascular condition that frequently goes undiagnosed by conventional diagnostic and treatment techniques until it reaches a more advanced stage. This challenge impedes the capacity to apply early detection and intervention measures. As a result, the creation of innovative and more accurate biomarkers is critically important. The study first recognizes genes associated with macrophages through single-cell analysis, investigating their functions. Subsequently, various machine learning approaches are utilized to identify significant regulatory genes related to macrophages. In addition, molecular docking studies are performed to evaluate the binding affinity of these crucial markers with therapeutics targeting atherosclerosis. The ImmuCellAI platform is also utilized to assess immune cell scores in atherosclerotic samples, aiding in the examination of connections between vital diagnostic markers and immune cells. Finally, the expression changes of the selected key genes are confirmed using qRT-PCR and Western blot methods. Through analyses at the single-cell level and differential assessments, we discovered 58 genes related to macrophages that exhibited differential expression. Functional evaluations indicated a strong correlation between these genes and the immune microenvironment. By conducting cluster analysis, we assessed how different subgroups of patients with atherosclerosis respond to immunotherapy. Utilizing techniques such as XGBoost, random forest, and the GOsemsim algorithm, we pinpointed five crucial diagnostic markers. Studies on molecular docking validated that these important markers could act as potential drug targets for atherosclerosis. Finally, our experimental analysis revealed a significant overexpression of these five diagnostic markers in tissues affected by atherosclerosis. This research introduces novel diagnostic indicators associated with macrophages in atherosclerosis and emphasizes their potential as targets for therapies related to the immune system.

## 1. Introduction

Atherosclerosis is a complex pathological condition primarily marked by lipid accumulation, inflammation, and endothelial dysfunction within arterial walls [1, 2]. These changes lead to blood vessel narrowing and stiffness, ultimately elevating the risk of subsequent cardiovascular events. Despite advancements in therapeutic strategies—such as statins to lower lipid levels and anti-inflammatory approaches—challenges in diagnosis and management persist [3]. Current biomarkers, including cholesterol levels and C-reactive protein, often lack the precision needed to accurately assess disease risk, monitor progression, or evaluate treatment efficacy. They frequently fail to fully capture the intricate pathophysiological mechanisms of atherosclerosis or account for individual variability [4]. Additionally, as a chronic and progressive disease, early detection remains difficult. Existing clinical tools are insufficient for identifying the condition at initial stages, leading to diagnoses often occurring only when advanced disease limits treatment options [5]. Therefore, the discovery of novel biomarkers is critical. Such markers could enable earlier identification of high-risk individuals and facilitate personalized interventions, ultimately improving clinical outcomes and quality of life. Moreover, these innovative biomarkers could significantly advance precision medicine by providing deeper insights into the molecular, environmental, and genetic factors underlying atherosclerosis.

Macrophages are crucial in the pathogenic processes associated with atherosclerosis, which is characterized as a chronic inflammatory condition. These immune cells engage in various pathways. Originating from circulating monocytes, they represent the primary white blood cell population found in atherosclerotic plaques [6]. Once recruited into the plaques, macrophages transform into foam cells by phagocytosing oxidized low-density lipoprotein (oxLDL), a hallmark of early lesion development [7]. In addition to their primary functions, macrophages play a crucial role in the advancement of diseases by facilitating lipid buildup and maintaining localized inflammation via the release of a range of inflammatory substances [8]. During different stages of atherosclerosis, macrophages exhibit diverse phenotypes and functions. For example, M1 macrophages are associated with proinflammatory responses, whereas M2 macrophages are linked to anti-inflammatory functions and tissue repair processes [9]. The metabolic condition of macrophages is closely linked to their functional roles. Research indicates that the glycolytic processes in these cells are deeply associated with their immune functions, as metabolic pathways play a crucial role in the progression of atherosclerosis [10]. Furthermore, autophagy in macrophages appears to exert a protective effect against atherosclerosis by reducing inflammation and apoptosis, in addition to aiding cholesterol efflux [11]. In the context of atherosclerosis treatment, macrophages represent promising therapeutic targets. Modulating their activity and phenotypic states can substantially impact disease progression. For example, strategies aimed at promoting M2 macrophage polarization and enhancing macrophage autophagy are believed to confer protective effects against atherosclerosis [12]. In summary, due to their central role in mediating inflammatory responses and driving the pathological development of the disease, macrophages are a critical focus for ongoing research and the development of targeted therapies for atherosclerosis.

In recent years, significant advancements in single-cell analysis technology have profoundly transformed the field of biomedicine [13, 14]. This innovative approach surpasses the limitations of traditional bulk sequencing by enabling detailed characterization of gene expression and cellular heterogeneity at the single-cell level. When combined with multiomics techniques, it allows for comprehensive mapping of complex biological networks across multiple layers, including the genome, transcriptome, and proteome [15, 16]. Such multidimensional investigations have substantially enhanced our understanding of disease mechanisms, opening new pathways for precision medicine. In this study, we integrated single-cell analysis with machine learning algorithms to systematically explore the functions of macrophage-related genes and their predictive value for immunotherapy responses in patients with atherosclerosis. Using XGBoost and random forest models, we evaluated the diagnostic relevance of these genes for atherosclerosis. Subsequently, experimental validation confirmed the expression levels of key biomarkers identified as potential diagnostic indicators for the disease.

## 2. Materials and Methods

### 2.1. Sample Collection

This study examined two atherosclerotic samples (GSM5577199 and GSM5577200) obtained from the GSE184073 dataset using single-cell resolution analysis. Additionally, RNA sequencing data and corresponding clinical information from the GSE100927 and GSE23746 datasets were integrated to enrich the analysis.

### 2.2. Single-Cell RNA Sequencing

We employed the Seurat package to construct data objects and filter out low-quality cells, ensuring that our analysis was based on high-quality data [17–19]. Our preprocessing followed a standardized protocol, focusing on key metrics such as gene counts, mitochondrial content, and cell expression levels. Specifically, we excluded genes detected in fewer than three cells and removed cells expressing fewer than 200 genes. To standardize UMI counts across samples, we applied a normalization method using a scaling factor of 10,000, followed by a log transformation to stabilize variance. Additionally, the ScaleData function from Seurat (v3.0.2) was used to further refine data quality for downstream analyses. To identify the main sources of variability, we selected the Top 10 most variable genes for principal component analysis (PCA), a technique effective for dimensionality reduction while preserving key features. We retained the first 11 principal components for visualization and clustering, as this captured essential structural information of the data. Cell clustering was performed using the FindClusters function with a resolution of 0.5, a parameter chosen to balance cluster granularity and interpretability, thereby revealing distinct cellular populations within the dataset.

### 2.3. Cluster Analysis

In our study, we employed the ConsensusClusterPlus R package (Version 1.54.0) to perform consensus clustering, allowing for the identification of up to six distinct clusters. To enhance the robustness of the results, we conducted a resampling procedure in which 80% of the dataset was randomly sampled with replacement, repeated 100 times. The clustering was performed using the hierarchical clustering algorithm (clusterAlg = “hc”) with the Ward.D2 linkage method, facilitating the detection of stable and meaningful data patterns. Following the consensus analysis, we utilized the pheatmap R package (Version 1.0.12) to generate cluster heatmaps of gene expression. To focus on the most informative genes, only those with a variance greater than 0.1 were included. When the number of target genes exceeded 1000, we ranked them based on their variance in descending order and selected the Top 25% with the highest variance for visualization. This approach ensured that the heatmaps emphasized the genes most likely to contribute significantly to the observed heterogeneity [20].

### 2.4. Functional Analysis

We used the KEGG REST API to obtain the most recent gene annotations related to KEGG pathways relevant to our background dataset. This step ensured that our analysis was grounded in up-to-date and accurate genetic information. The identified genes were then systematically mapped onto this dataset, enabling a more comprehensive functional analysis.

For the enrichment analysis, we employed the R package clusterProfiler (Version 3.14.3), which facilitated the identification of enriched KEGG pathways and provided insights into the biological significance of our gene set within these pathways. In addition, for Gene Ontology (GO) analysis, we utilized annotations from the org.Hs.eg.db package (Version 3.1.0) as our reference background. This reliable resource ensured that our enrichment results were based on accurate and current gene annotations. Using clusterProfiler again, we integrated our gene list into this background to perform a detailed GO enrichment analysis, revealing functional insights and relationships among the genes in our study [21, 22].

### 2.5. Analysis of Immune Cell Infiltration

The ImmuCellAI algorithm calculates the abundance of 24 types of immune cells by examining gene expression profiles obtained from RNA-Seq or microarray datasets [23]. For performing immune infiltration analysis, you need to upload the standardized gene expression matrix to ImmuCellAI and utilize the Wilcoxon rank-sum test for comparisons between groups [24].

### 2.6. Molecular Docking of Key Genes With Aspirin

To evaluate the binding affinity of crucial genes with drugs used for atherosclerosis treatment, we conducted an analysis using molecular docking techniques. The incorporation of the CB-Dock2 platform significantly enhanced our research, allowing us to assess the genes' affinity for the medications through the application of the Vina score [25]. A Vina score below −5.0 kcal/mol is commonly acknowledged as a sign of stronger binding interactions between these elements [26].

### 2.7. Quantitative Real-Time PCR (qRT-PCR)

Total RNA was extracted from atherosclerotic tissue samples using TRIzol reagent, followed by cDNA synthesis with HiScriptII SuperMix. qRT-PCR was performed on an ABI7500 system using SYBR Green MasterMix. The amplification protocol consisted of 45 cycles, beginning with an initial incubation at 94°C for 10 min and then 10 s at 94°C and 45 s at 60°C per cycle. GAPDH was used as the internal control. The primer sequences for the target genes are listed below: TYROBP (Forward: GGACTTGAACCCTGCAGCAG, Reverse: TACGCTGTTTCCGGGTCGCT), CTSB (Forward: AGTGGAGAATGGCACACCCTA, Reverse: AAGAAGCCATTGTCACCCCA), PYCARD (Forward: TGGATGCTCTGTACGGGAAG, Reverse: CCAGGCTGGTGTGAAACTGAA), LAPTM5 (Forward: GCGTCTTGTTGTTCATCGAGC, Reverse: CGATCCTGAGGTAGCCCAT), SLC15A3 (Forward: ACATCAACAATTGCCGGATGGACC, Reverse: ATAGCGTCCAGCGATCCAGACAAA), and GAPDH (Forward: CGGAGTCAACGGATTTGGTCGTAT, Reverse: AGCCTTCTCCATGGTGGTGAAGAC).

### 2.8. Western Blot

For the immunoblotting procedure, cells and tissues were lysed for a duration of 30 min at 4°C using Lysis Reagent (SolarbioR0020, China), which was supplemented with a mixture of phosphate and protease inhibitors. Subsequent to this step, the lysates underwent SDS polyacrylamide gel electrophoresis (PAGE) at a concentration of 10% and were then transferred onto a nitrocellulose membrane (PerkinElmer, Waltham, Massachusetts). The membrane was blocked with 5% BSA in PBS and incubated overnight at 4°C with primary antibodies. This was followed by a 1-h incubation at room temperature with the corresponding secondary antibodies [27]. The results were visualized using the BIO-RAD ChemiDoc XRS+ system. In this study, the antibodies employed included TYROBP (28138-1-AP, 1:500), CTSB (12216-1-AP, 1:2000), PYCARD (10500-1-AP, 1:3000), LAPTM5 (A17995, 1:500), and SLC15A3 (20866-1-AP, 1:1000).

### 2.9. Statistical Analyses

The assessment of statistical differences between the two groups was conducted utilizing a *T*-test. The Spearman method was employed for the correlation analysis. A *p* value of less than 0.05 was deemed statistically significant.

## 3. Results

### 3.1. Single-Cell Transcriptomics Analysis Identified Macrophage-Related Subpopulations

**Figure 1:**
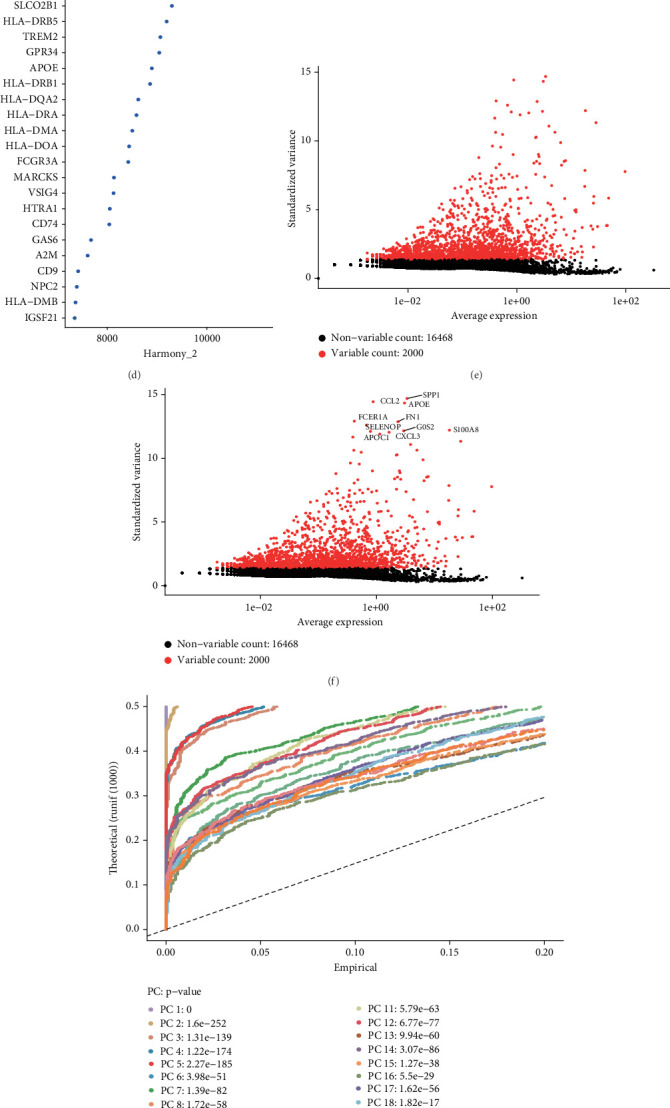
Identification of marker genes for macrophage. (a) Assessment of scRNA-seq quality across various sub-populations of cells. (b–d) Visual depictions of PCA analysis conducted after the integrated elimination of batch effects. (e, f) Identification of highly variable genes executed postcount following batch removal. (g–i) Application of the UMAP method to categorize atherosclerosis samples. (j) Cell distribution map in different groups.

Our study began with the analysis of two atherosclerosis samples obtained from the GSE184073 dataset, implementing rigorous quality control criteria for cell selection. Each cell was required to contain at least 200 and no more than 5000 RNA molecules, with mitochondrial RNA accounting for no more than 15% of total RNA content (Figure 1a). Following this, we utilized the Harmony framework to identify highly variable genes from the curated dataset and performed batch effect correction using these identified gene sets (Figures 1b, 1c, and 1d). Variance analysis revealed 10 genes with significant differential expression across the cell populations: SPP1, CCL2, APOE, FCER1A, FN1, SELENOP, G0S2, S100A8, APOC1, and CXCL3 (Figure 1e,f). Further, single-cell clustering divided the two atherosclerotic samples into six distinct cell types: platelets, macrophages, natural killer (NK) cells, B cells, monocytes, and T cells (Figures 1g, 1h, and 1i). Additionally, we provided a visualization illustrating the distribution of these cell populations across different groups (Figure 1j).

### 3.2. Identify Macrophage-Related Differential Genes

**Figure 2:**
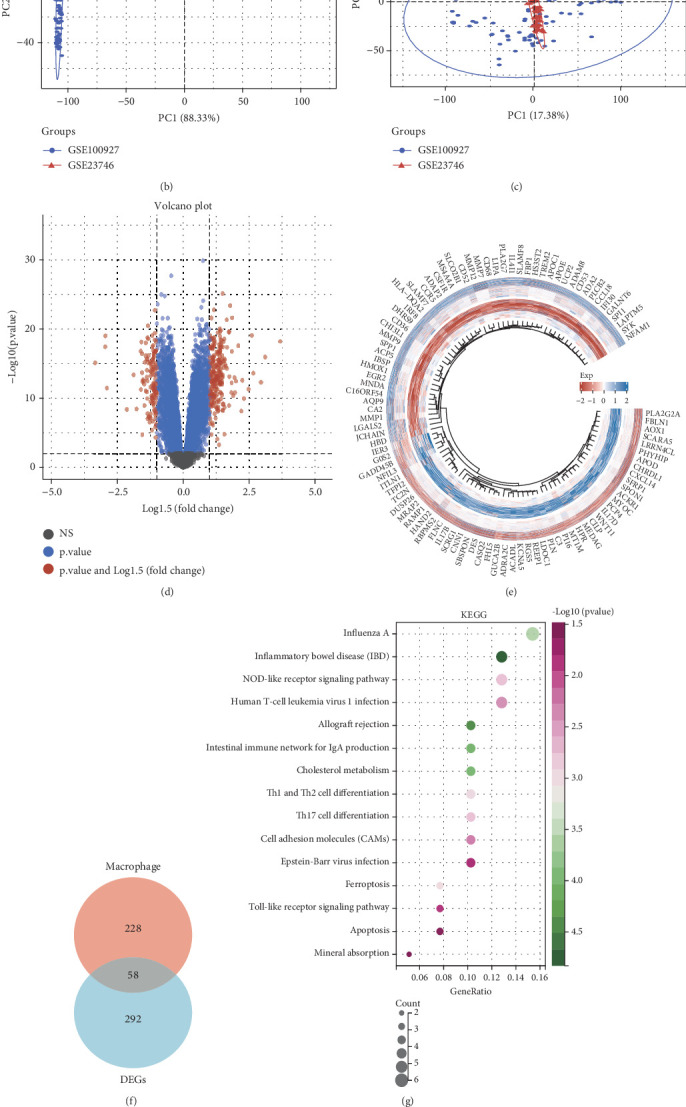
Analysis of functions related to differential genes associated with macrophage. (a) Examination of correlations among various cell populations and distinct pathways. (b) PCA findings prior to eliminating batch effects across different datasets. (c) PCA outcomes subsequent to the removal of batch effects from various datasets. (d, e) Assessment of differences. (f) The Venn diagram illustrates the overlap between macrophage-related genes and genes that are differentially expressed. (g, h) Analysis of gene enrichment.

Building on our single-cell analysis, we observed a positive correlation between genes and pathways linked to macrophages, including angiogenesis, collagen production, and the inflammatory response (Figure 2a). To identify potential biomarkers associated with atherosclerosis development, we retrieved two additional datasets (GSE100927 and GSE23746) from the GEO database, encompassing samples from 145 atherosclerosis patients and 54 healthy controls. These datasets were standardized and integrated to create a unified high-throughput sequencing dataset. PCA conducted before and after batch effect removal demonstrated successful data integration, enabling reliable downstream analysis (Figure 2b,c). Differential expression analysis using the limma package in R identified genes significantly altered between patient and control samples (Figure 2d,e). Intersection analysis pinpointed 58 macrophage-related genes differentially expressed in atherosclerosis (Figure 2f). KEGG pathway enrichment analysis showed that these genes participate in pathways like Influenza A, inflammatory bowel disease, and NOD-like receptor signaling. Additionally, GO enrichment analysis suggested that these genes are linked to immune-related processes, encompassing immune responses, activities of the immune system, and inflammatory reactions (Figure 2g,h). Collectively, our results suggest that the differentially expressed macrophage-related genes play pivotal roles in shaping the immune microenvironment of atherosclerosis.

### 3.3. Consistent Clustering Analysis Based on Macrophage-Related Genes

**Figure 3:**
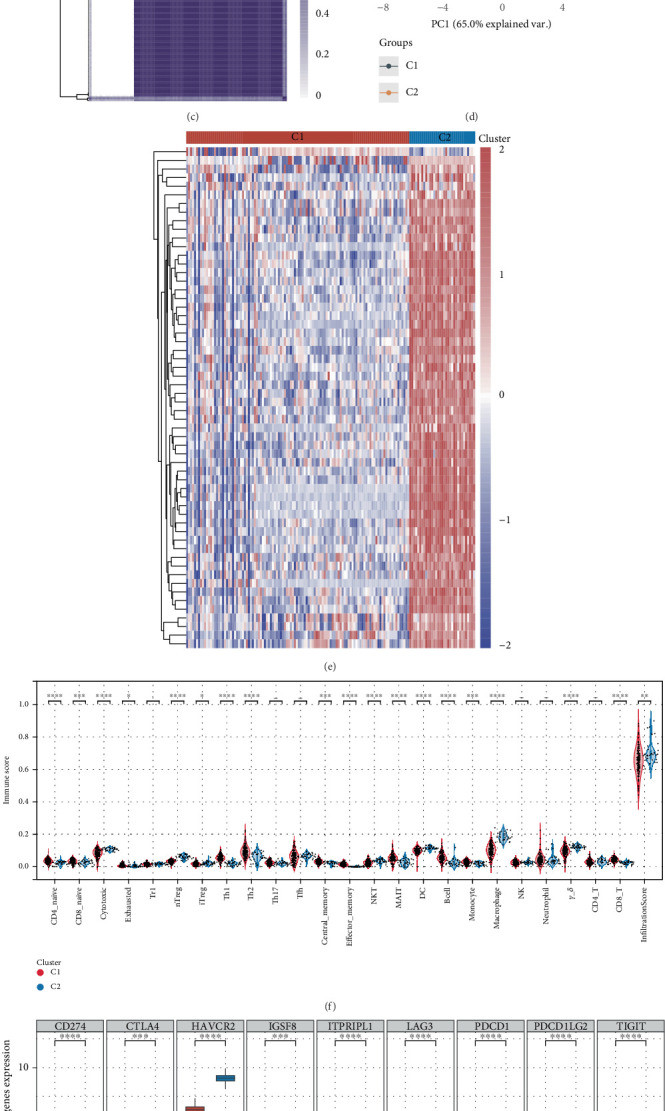
Macrophage-related genes are related to immune therapy in patients with atherosclerosis. (a) Cumulative distribution graph. (b) Area beneath the distribution graph. (c) Heatmap showcasing clustering. (d) Consistency of sample clustering. (e) Expression levels of macrophage-associated genes across two clusters. (f) Variations in immune cell infiltration levels across the different clusters. (g) Differences in the expression levels of immune checkpoints among the various clusters. (h) Assessment of the responsiveness of the two clusters treated with immunosuppressive agents.

A consensus clustering analysis was conducted using the expression profiles of macrophage-related genes derived from the GSE100927 and GSE23746 datasets. To identify the ideal number of clusters, researchers established the *K* value that produced the lowest “fuzzy clustering ratio,” a method commonly accepted in consensus clustering studies. The PAC index, which quantifies central tendency, is defined as the consensus index within the interval (*u*1, *u*2) ∈ [0, 1], where *u*1 approaches 0 and *u*2 nears 1 (for instance, *u*1 = 0.2 and *u*2 = 0.8). A lower PAC score indicates a smoother transition in the central section, revealing fewer inconsistencies throughout the shuffled clustering processes. The cumulative distribution curve, along with the area beneath it, demonstrated that the maximum average consistency within the group occurred when *K* equaled 2. Furthermore, a clustering heatmap was generated for *K* = 2 (Figures 3a, 3b, 3c, and 3d). In this scenario, atherosclerotic samples were classified into two groups: Cluster 1, which included 112 samples, and Cluster 2, which contained 33 samples (Figure 3e). Given the strong correlation observed between macrophage-related genes and the immune microenvironment in patients with atherosclerosis, we also calculated the infiltration scores of 24 immune cells in the affected samples using the ImmuCellAI algorithm. Among the immune cells examined from Clusters 1 and 2, only six displayed no significant differences in their infiltration levels (Figure 3f). Additionally, we evaluated the differences in gene expression of immune checkpoint-related genes across Clusters 1 and 2, uncovering considerable variations among all immune checkpoint-related genes included in our analysis (Figure 3g). In summary, we assessed how patients from various clusters responded to immune checkpoint inhibitor therapy. Our results indicate that those in Cluster 1 showed better treatment results after receiving therapy with immune checkpoint inhibitors (Figure 3h).

### 3.4. Functional Analysis Between Different Clusters

**Figure 4:**
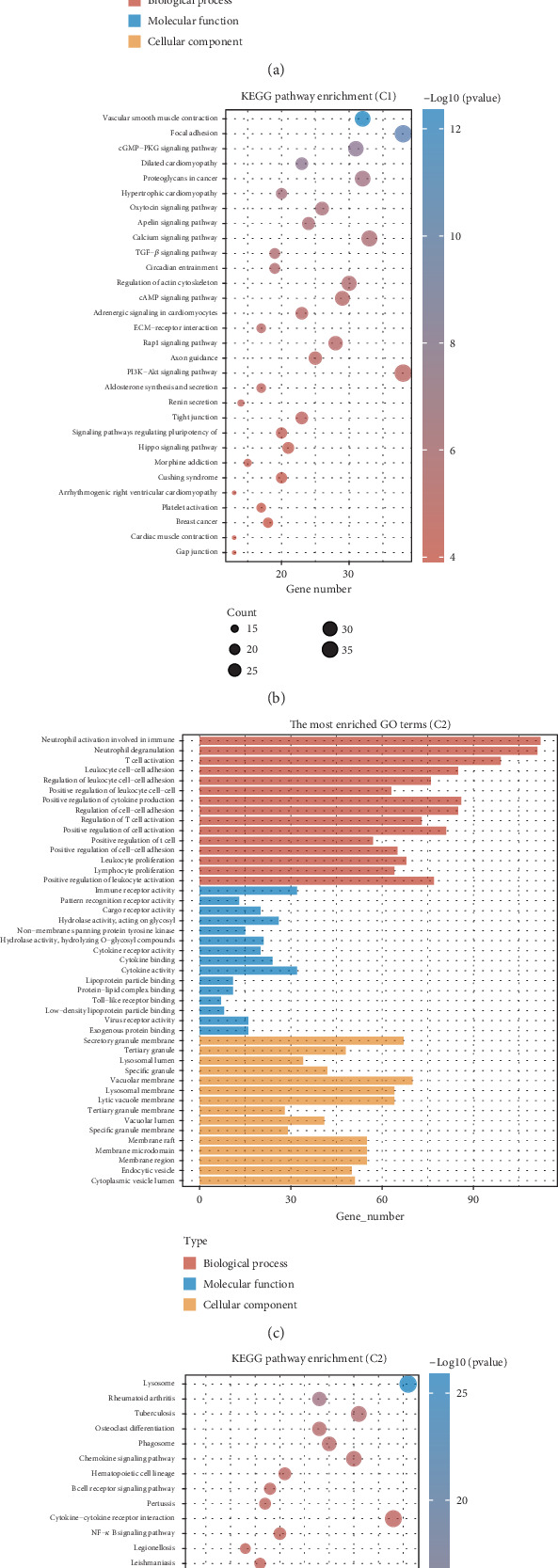
Functional analysis. (a, b) Relevant pathways of KEGG and GO analysis Cluster 1. (c, d) Relevant pathways of KEGG and GO analysis Cluster 2.

To comprehensively analyze the differences in immune infiltration and immunotherapy responses across the various clusters, we performed KEGG and GO enrichment analyses. GO analysis revealed that samples in Cluster 1 are primarily associated with processes related to the muscle system, including muscle contraction, muscle organ development, actin binding, structural components of muscle, and elements of the extracellular matrix, contractile fibers, myofibrils, and sarcomeres. In contrast, Cluster 2 is enriched for processes involved in immune activation, such as neutrophil degranulation, neutrophil activation in immune responses, T cell activation, immune receptor functions, pattern recognition receptor activity, cargo receptor functions, and components of secretory granule membranes, tertiary granules, and lysosomal lumens. KEGG pathway analysis showed that Cluster 1 is associated with pathways including focal adhesion, the PI3K−Akt signaling pathway, calcium signaling, the cGMP−PKG pathway, and regulation of the actin cytoskeleton. Conversely, Cluster 2 is linked to lysosomal activities, cytokine-receptor interactions, chemokine signaling pathways, and B cell receptor signaling (Figures 4a, 4b, 4c, and 4d).

### 3.5. Machine Learning Algorithm Identifies Key Genes

**Figure 5:**
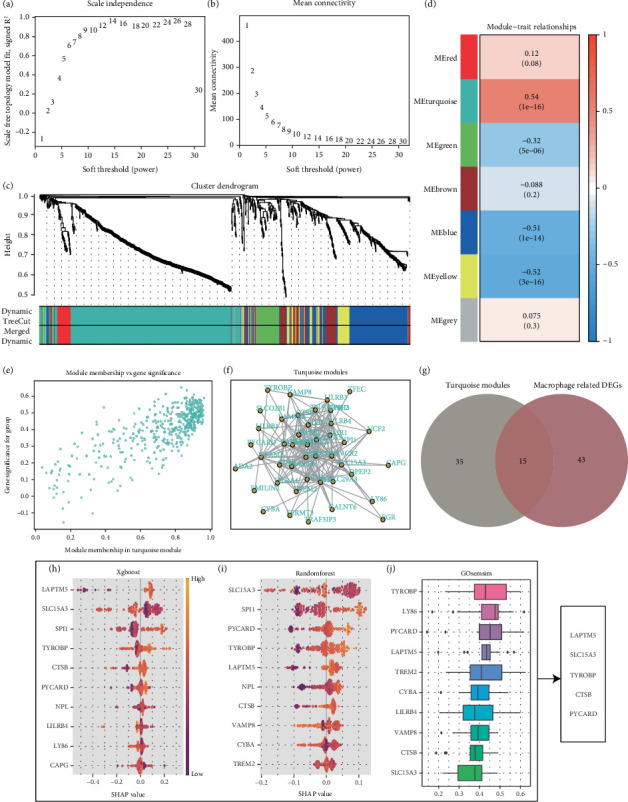
TYROBP, CTSB, PYCARD, LAPTM5, and SLC15A3 are considered to be key diagnostic markers of atherosclerosis. (a, b) The ideal power for soft-thresholding was determined to be 9. (c) Construction of a weighted coexpression network was carried out utilizing the chosen power values. (d) A heatmap displaying the associations between trait modules was generated. (e) Scatter plot of the association between specific traits and module genes. (f) The interaction network diagram of genes within the module. (g) Venn diagram. (h–j) Machine learning algorithms identify key diagnostic genes and take the intersection.

To identify potential diagnostic biomarkers for atherosclerosis, we performed weighted gene coexpression network analysis (WGCNA) using the GSE100927 and GSE23746 datasets. To ensure that the network conformed to a scale-free topology, we selected a soft-thresholding power of 9 for constructing the adjacency matrix (Figure 5a,b). Using this parameter, a weighted coexpression network was built, and all genes were grouped into seven distinct modules (Figure 5c,d). We then calculated the Pearson correlation coefficients and associated *p* values between each module's eigengene and specific traits, revealing that the turquoise module had the strongest correlation with a coefficient of 0.54 (Figure 5e). From this module, the Top 50 genes were selected to construct an interaction network (Figure 5f). By cross-referencing these 50 hub genes with known macrophage-associated genes, we identified 15 candidate diagnostic markers relevant to macrophages (Figure 5g). Further, using machine learning algorithms such as XGBoost, random forest, and the GOsemsim similarity measure, we pinpointed the most significant diagnostic genes: TYROBP, CTSB, PYCARD, LAPTM5, and SLC15A3 (Figures 5h, 5i, and 5j).

### 3.6. Expression and Correlation Analysis of Key Diagnostic Biomarkers

**Figure 6:**
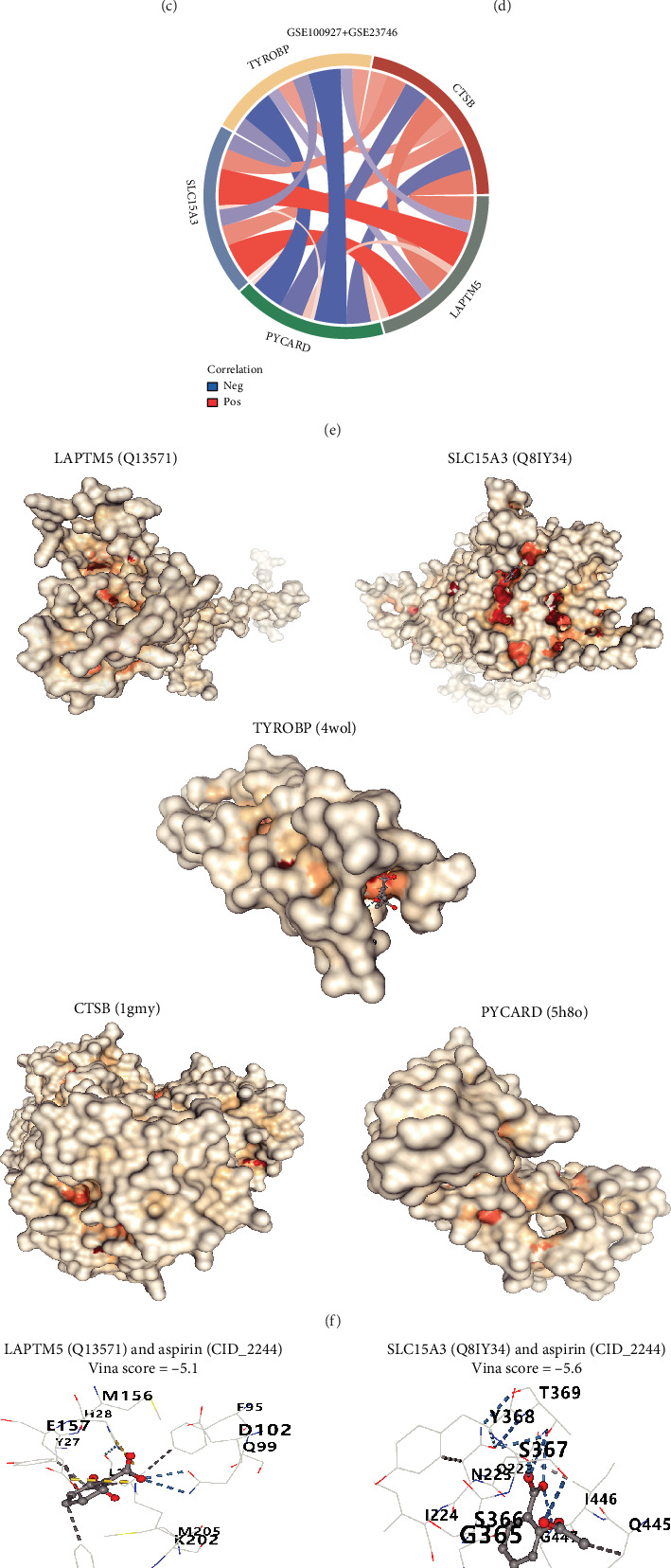
TYROBP, CTSB, PYCARD, LAPTM5, and SLC15A3 can be used as treatment targets for atherosclerosis. (a) Expression analysis of key diagnostic markers of atherosclerosis. (b) Predictive value of TYROBP, CTSB, PYCARD, LAPTM5, and SLC15A3 in the diagnosis of atherosclerosis. (c–e) Correlation analysis of TYROBP, CTSB, PYCARD, LAPTM5, and SLC15A3. (f, g) The molecular docking of TYROBP, CTSB, PYCARD, LAPTM5, and SLC15A3 with aspirin.

This study began with analyzing the GSE100927 and GSE23746 datasets to evaluate the expression levels of five key genes—TYROBP, CTSB, PYCARD, LAPTM5, and SLC15A3—in atherosclerosis-affected samples compared to normal controls. The analysis revealed a significant upregulation of these genes in atherosclerotic samples relative to healthy tissues (Figure 6a). To assess their potential as diagnostic biomarkers, receiver operating characteristic (ROC) curve analysis was performed, yielding AUC values of 0.817 for TYROBP, 0.757 for CTSB, 0.803 for PYCARD, 0.736 for LAPTM5, and 0.692 for SLC15A3 (Figure 6b). These results suggest that these genes may serve as promising indicators for diagnosing atherosclerosis. Additionally, an interaction network among these five genes was visualized using a chord diagram (Figures 6c, 6d, and 6e). In order to investigate possible therapeutic uses, studies involving molecular docking were performed to assess how well these genes bind with aspirin, which is a frequently utilized therapy for atherosclerosis. The results from the docking analysis indicated that all five genes exhibited strong binding interactions with aspirin (Figure 6f,g).

### 3.7. Analysis of Immune Cell Infiltration

**Figure 7:**
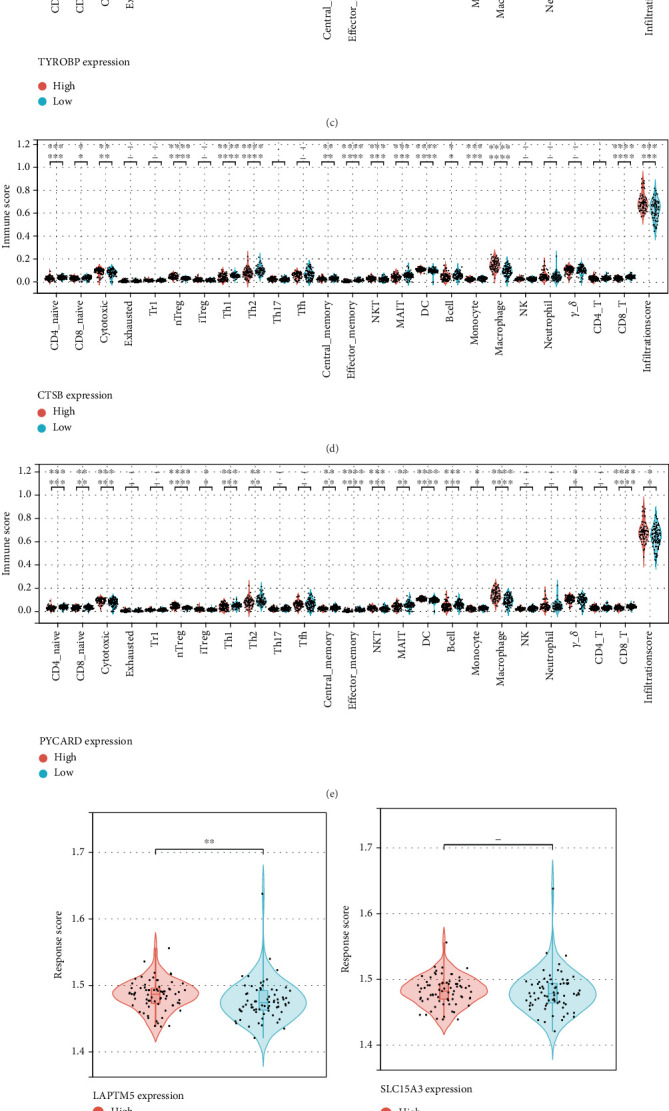
The relationship between key identified genes and immune cells. (a–e) The differences in the infiltration levels of immune cells among different groups. (f–j) The responses of patients to immune checkpoint inhibitor therapy in different groups.

Recent clinical and experimental studies suggest that immune mechanisms play a role in the progression of atherosclerosis. This insight prompted us to explore the relationship between key molecular markers and immune cell infiltration within atherosclerotic samples. Using the datasets GSE100927 and GSE23746, we categorized samples based on high and low expression levels of LAPTM5. Our analysis revealed significant differences in the infiltration levels of 17 immune cell types between the high and low LAPTM5 expression groups (Figure 7a). Similarly, comparing groups with high and low expression of SLC40A1 uncovered variations in 15 immune cell types (Figure 7b). Analysis of TYROBP expression levels demonstrated differences in 16 immune cell infiltrates between high and low expression groups (Figure 7c), while groups stratified by CTSB expression showed differences in 15 immune cell types (Figure 7d). Notably, significant differences were also observed in the infiltration of 17 immune cell types when comparing high versus low PYCARD expression groups (Figure 7e). Finally, we assessed responses to immune checkpoint inhibitor therapy across these groups, indicating that the expression levels of LAPTM5, CTSB, and PYCARD may serve as potential indicators of patient response to immunotherapy (Figures 7f, 7g, 7h, 7i, and 7j).

### 3.8. Expression Verification of Key Genes for Atherosclerosis

**Figure 8:**
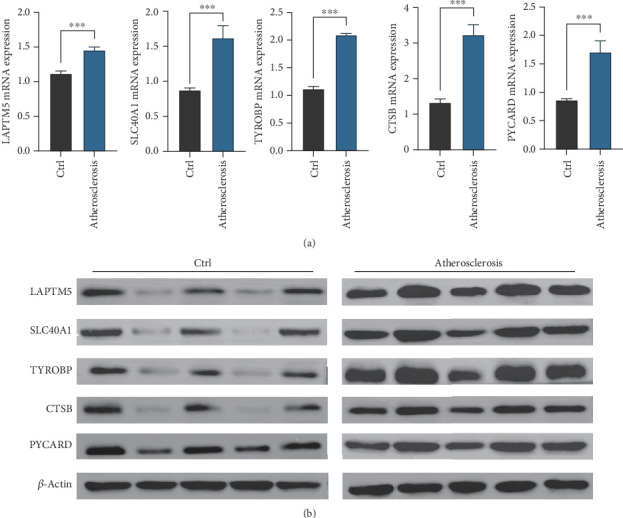
Validation of the expression of key diagnostic genes. (a) Verify the expression differences of TYROBP, CTSB, PYCARD, LAPTM5, and SLC15A3 through qRT-PCR. (b) Verify the expression differences of TYROBP, CTSB, PYCARD, LAPTM5, and SLC15A3 through Western blot.

Our study highlights the important roles of LAPTM5, SLC40A1, TYROBP, CTSB, and PYCARD in the context of atherosclerosis. To further validate these findings, we conducted qRT-PCR and Western blot analyses on serum samples from five individuals diagnosed with atherosclerosis and five healthy controls. The results demonstrated significantly higher expression levels of LAPTM5, SLC40A1, TYROBP, CTSB, and PYCARD in the serum of atherosclerosis patients compared to the healthy controls (Figure 8a,b). These findings reinforce our previous data and suggest that these markers hold promise as potential diagnostic indicators for atherosclerosis.

## 4. Discussion

In modern society, atherosclerosis is a widespread and serious cardiovascular disease that poses significant challenges for diagnosis and management. Conventional diagnostic methods predominantly rely on imaging techniques and blood biochemical tests [28, 29]. However, these approaches often detect abnormalities only after the disease has reached an advanced stage, limiting the potential for early intervention. Ideally, biomarkers should enable early detection, monitor disease progression, and inform personalized treatment strategies. In the context of precision medicine, the search for biomarkers with high specificity and stability is crucial—not only to enhance diagnostic accuracy but also to identify key therapeutic targets. Such biomarkers can facilitate the development of new drugs and innovative treatment approaches, ultimately improving the prevention and control of cardiovascular diseases.

Macrophages are essential in both the development and advancement of atherosclerosis, especially within its immune microenvironment. This condition is a long-lasting inflammatory disease defined by lipid accumulation in the arterial walls, which activates immune and inflammatory reactions [30]. The versatility and adaptability of macrophages allow them to display various functional states in response to different stimuli in the microenvironment, including proinflammatory M1 and anti-inflammatory M2 types [31]. In the realm of atherosclerotic lesions, the polarization state of macrophages plays a crucial role in the advancement and stability of plaques. M1 macrophages exacerbate inflammation by releasing proinflammatory cytokines, whereas M2 macrophages contribute to plaque stability by promoting tissue repair and fibrosis [32]. Additionally, the metabolic reprogramming of macrophages is crucial in the development of atherosclerosis. Studies suggest that changes in macrophage glycolysis and oxidative phosphorylation pathways can impact their functional state, ultimately affecting lesion progression [33]. The functional capabilities of macrophages are intricately connected to their metabolic state in atherosclerosis, which presents new avenues for therapeutic strategy development [34]. Beyond merely mediating inflammatory responses, macrophages also play an essential role in lipid metabolism and foam cell formation in atherosclerosis. By altering the functional state of these cells, it is possible to influence the progression of atherosclerosis, potentially establishing them as a novel therapeutic target for the disease [35]. Consequently, a comprehensive exploration of the mechanisms through which macrophages affect atherosclerosis is vital for the advancement of immunomodulatory therapies for this condition.

Recently, the idea of precision medicine has attracted considerable interest among the medical community, fueling initiatives to more precisely classify individual patients. This methodology improves our grasp of disease mechanisms and opens up avenues for tailored treatment strategies. By leveraging precision medicine, researchers can identify pathogenic pathways and prognostic features specific to different patient subgroups, ultimately enabling more precise and effective therapies. In our study, we classified atherosclerotic patients into two subtypes based on macrophage-related gene expression. Our findings revealed notable differences in immunotherapy responses between patients in Cluster 1 and those in Cluster 2. Functional analysis indicated distinct regulatory pathways underpinning these two groups. Using XGBoost and random forest algorithms, we identified key diagnostic genes associated with atherosclerosis, and by integrating the GOsemsim algorithm, we pinpointed five critical genes. Molecular docking studies demonstrated that these macrophage-associated genes exhibit strong binding affinity with aspirin, suggesting they could serve as promising drug targets for atherosclerosis. Moreover, we validated the differential expression of these five genes through experimental methods, reinforcing their potential clinical significance.

## 5. Conclusion

In our study, we utilized several machine learning algorithms to determine that LAPTM5, SLC40A1, TYROBP, CTSB, and PYCARD, which are related to macrophage genes, can act as diagnostic indicators for individuals with atherosclerosis and are linked to immune infiltration in this disease.

## Data Availability

The data that support the findings of this study are available from the corresponding authors upon reasonable request.
